# Amplitude- and frequency-dependent activation of layer II/III neurons by intracortical microstimulation

**DOI:** 10.1016/j.isci.2023.108140

**Published:** 2023-10-06

**Authors:** Guangying K. Wu, Yasaman Ardeshirpour, Christina Mastracchio, Jordan Kent, Michael Caiola, Meijun Ye

**Affiliations:** 1 Division of Biomedical Physics, Office of Science and Engineering Laboratories, Center for Devices and Radiological Health, Food and Drug Administration, Silver Spring, MD 20993, USA; 2 Scientific Publications Department, Society for Neuroscience, Washington DC, USA

**Keywords:** Neuroscience, Cell biology

## Abstract

Intracortical microstimulation (ICMS) has been used for the development of brain machine interfaces. However, further understanding about the spatiotemporal responses of neurons to different electrical stimulation parameters is necessary to inform the design of optimal therapies. In this study, we employed *in vivo* electrophysiological recording, two-photon calcium imaging, and electric field simulation to evaluate the acute effect of ICMS on layer II/III neurons. Our results show that stimulation frequency non-linearly modulates neuronal responses, whereas the magnitude of responses is linearly correlated to the electric field strength and stimulation amplitude before reaching a steady state. Temporal dynamics of neurons’ responses depends more on stimulation frequency and their distance to the stimulation electrode. In addition, amplitude-dependent post-stimulation suppression was observed within ∼500 μm of the stimulation electrode, as evidenced by both calcium imaging and local field potentials. These findings provide insights for selecting stimulation parameters to achieve desirable spatiotemporal specificity of ICMS.

## Highlights

• ICMS frequency non-linearly modulates magnitude and dynamic of neuronal responses
• Magnitude of responses is linearly related to E-field and stimulation amplitude
• Response dynamics are related to the distance to stimulation electrode
• Post-stimulation suppression was observed within 500 μm of stimulation electrode

## Graphical abstract

**Figure:**
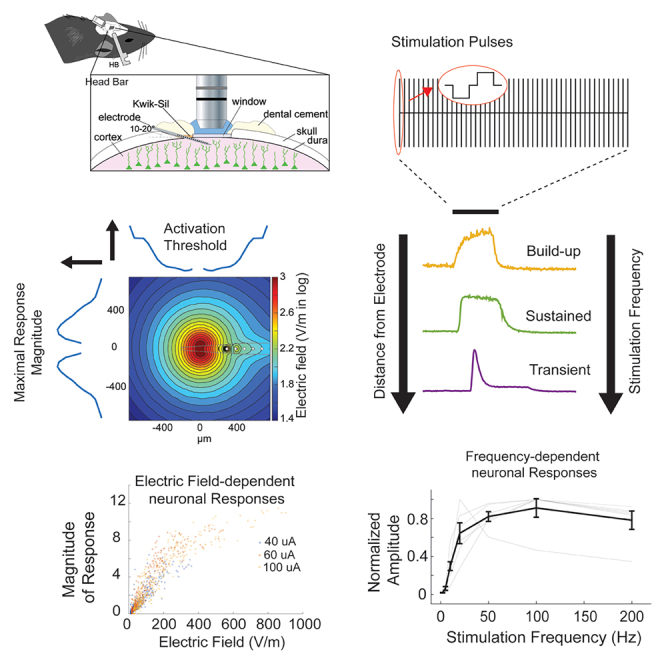

## Introduction

Cortical microelectrode implants have garnered increasing interests as therapeutic devices for neurological disorders in recent years.^1^^,^^2^^,^^3^ In addition to recording neuronal activity for decoding patients’ perception or intention in a brain-computer interface (BCI) system, many of these implants are being investigated for cortical mapping and sensorimotor restoration through electrical stimulation, known as intracortical microstimulation (ICMS).^4^^,^^5^^,^^6^^,^^7^^,^^8^ These electrical stimulations via penetrative microelectrodes bypass the blood-brain-barrier and directly alter the neuronal activity in specific brain regions, providing a high spatiotemporal resolution.

The effectiveness of these electrical stimulations in providing sensory feedback or restoring perception is currently being investigated in human clinical trials. It was noted that the sensation features, e.g., sensory perception intensity, spatial discrimination, etc., can be finely tuned by adjusting the stimulation parameters.^4^^,^^5^^,^^7^^,^^8^^,^^9^^,^^10^^,^^11^^,^^12^ However, different results were observed: some studies showed a linear positive relationship between stimulation amplitude and magnitude of sensation up to the tested maximal stimulation intensity (16 nC per phase [ph]),^4^^,^^5^ whereas others demonstrated a plateau effect above 12 nC/ph.^4^ Schmidt et al. reported that 200 Hz stimulation always elicited equal or higher intensity of phosphene perception compared with 100 Hz stimulation when stimulation current amplitude and pulse duration remain the same,^7^ whereas Hughes et al. noted that the effect of stimulation frequency on the sensory perception intensity depends on electrode and stimulation amplitude.^6^ These variabilities indicate uncertainties in the design of optimal stimulation interventions and highlight the need for further understanding of the spatiotemporal relationship between neuronal responses and electrical stimulation parameters.

Recently, two-photon microscopy (TPM) calcium imaging was employed to probe the cellular mechanisms of ICMS.^13^^,^^14^^,^^15^^,^^16^^,^^17^^,^^18^ These studies elegantly characterized the spatial and temporal dynamics of neuronal responses to different stimulation paradigms at the cellular level and provided some insights into the inconsistent observations in different clinical and animal studies. However, these studies conservatively restricted stimulation amplitude at very low amplitude. They were unable to resolve the dilemma of how to maximize therapeutic effects of stimulation while minimizing damaging effects. An investigation of stimulation parameter domains in a wider range is needed to inform the effectiveness and safety of ICMS for clinical use.

Conventionally, the Shannon equation, which included charge density and charge per phase as determinants, is utilized to define the boundary between “safe” and “damaging” levels of electrical stimulation from macroelectrodes.^19^ However, the thresholds determined by the Shannon equation for macroelectrodes cannot be directly applied to stimulation from microelectrodes (electrodes with a surface area <2000 μm^2^), due to differences in the spatial charge distribution.^20^ A recent review of accumulating data suggested a safety threshold of 4 nC/ph for microstimulation.^21^ Nevertheless, mounting evidence demonstrates the sensory or motor restoration threshold for ICMS is in the range of 3–10 nC/ph with a stimulation frequency between 25 and 300 Hz, and the sensation features can be finely tuned by adjusting the stimulation intensity up to 20 nC/ph. Apparently, the 4 nC/ph safe limit restricts ICMS devices from achieving its optimal effectiveness.

Another limitation of the 4 nC/ph threshold as a safety reference is that it only considers charge per phase. Electrode geometry, stimulation pulse shape, and source location all influence the electrical field (E-field) applied to the neurons, which in turn affects the neuronal responses to electrical stimulation.^22^ Our current knowledge about the safety and effectiveness of electrical stimulation is largely derived from conventional needle-shaped electrodes, e.g., Utah array. In recent years, novel film microelectrodes with a high charge capacity are being designed to provide high spatial resolution for neural stimulation.^17^^,^^23^^,^^24^ Further investigation is needed to generalize the results obtained from needle-shaped electrodes for evaluating these novel film electrodes. As externally applied E-field plays a key role in neuron activation, establishing a relationship between external E-field and neuronal responses may facilitate the generalization of results to different types of electrodes.

To better inform the safe and effective design of ICMS therapy, this study employed *in vivo* electrophysiological recording, two-photon calcium imaging, and E-field simulation to capture the spatiotemporal profile of electrical stimulation and neuronal activity from Thy1-GCaMP6s mice. The neuronal activation with respect to stimulation amplitude, frequency, spatial distribution, and local E-field was examined. Specifically, we quantified the calcium signal strengths of whole fields and individual neurons, and the number of activated neurons in response to stimulation amplitude (1–100 μA) and frequency (2–200 Hz), within 1 mm radius from stimulation electrodes. Thus, we were able to define the effective activation and saturation thresholds for neurons at different locations. We also examined the temporal responses of neurons to stimulation amplitude and frequency, which can provide insight on how different parameters can affect the temporal features of stimulation-induced sensation.

In addition to the effect during stimulation, we examined the post-stimulation electrophysiological properties of neuronal activation. This effort was intended to bridge the gap of a lack of real-time biomarkers for *in vivo* quantification of electrical stimulation effect. Our previous study of brain injury indicates that electrophysiological changes can occur with minimal neural tissue damage,^25^ suggesting the potential of electrophysiology-based biomarkers as proxies to reveal the impact of electrical stimulation. Finally, we simulated E-field strengths around TPM imaged neurons, aiming to establish the spatiotemporal relationship between external E-field and neuronal activation.

## Results

**Figure 1:**
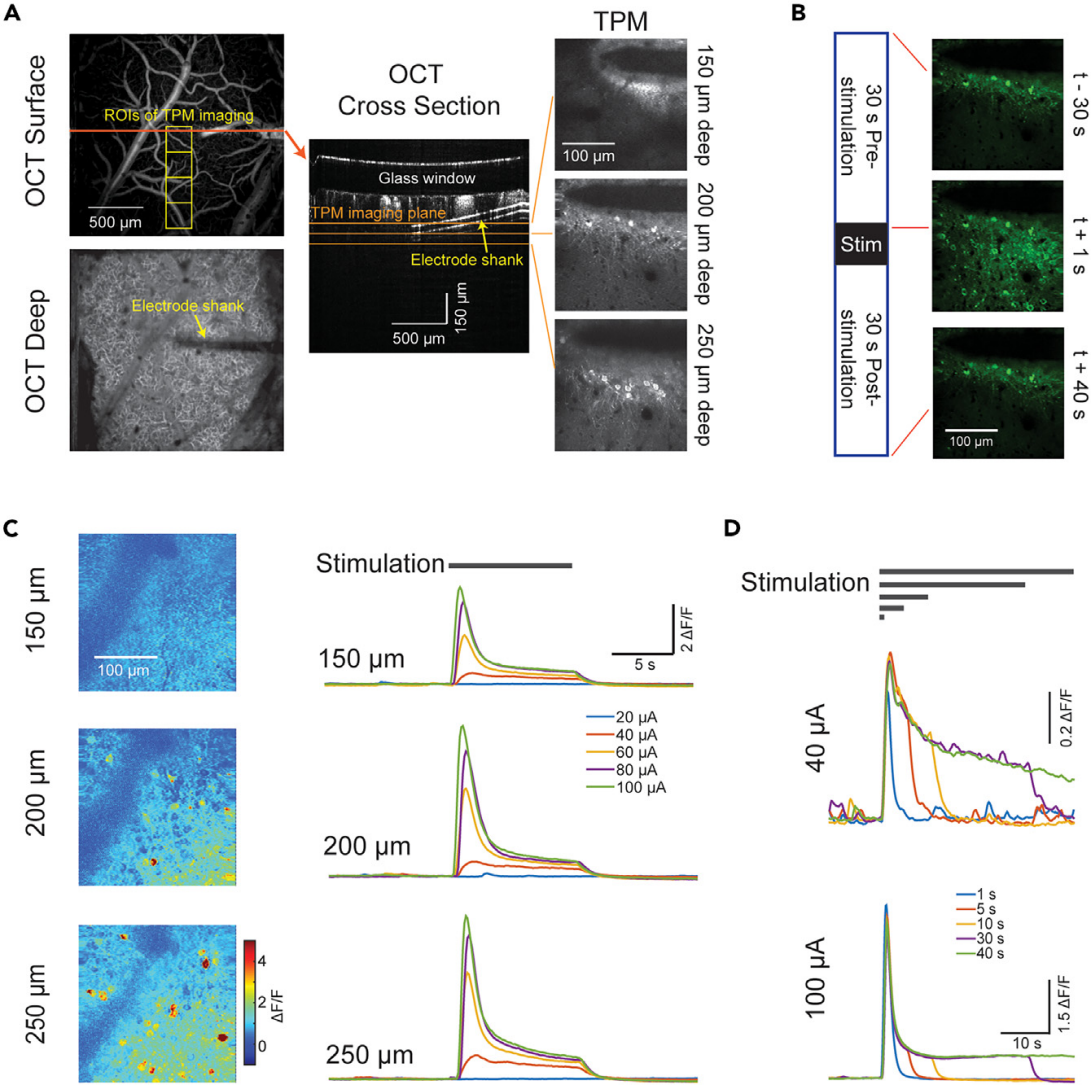
Experimental paradigm (A) OCT images at cortical surface and 300 μm deep (left, yellow squares indicating the regions for TPM), cross-section view from OCT imaging (middle) showing the track of the electrode shank. TPM images at different cortical depths. (B) TPM images of calcium signals from neurons approximal to the electrode (shadow seen in the upper area of each frame), before (top), during (middle), and after stimulation (bottom) at 30 μA and 50 Hz for 10 s. (C) Heat maps of amplitudes of full-field calcium signals evoked by stimulation (left) and the example calcium signal traces evoked by different current amplitudes at different cortical depths (right). (D) Calcium signals evoked by different durations (gray horizontal bars, 1 s, 5 s, 10 s, 30 s, and 40 s) of stimulation at two current amplitudes. The amplitude of responses is minimally affected by the duration of stimulation.

To investigate neuronal responses to electrical stimulation, single-shank, 16-channel, Michigan-style microelectrode arrays (A1X16-3mm-50-177-CM16LP or A1X16-3mm-100-703-CM16LP, NeuroNexus, Ann Arbor, MI) were implanted with an angle of 10–20° relative to the brain surface into the layer 2/3 somatosensory cortices of the transgenic mice expressing calcium indicators (Thy1-GCaMP6s) in excitatory neurons. Optical coherence tomography (OCT) imaging was performed to identify the tip of the probe and estimate the distance between the stimulating electrodes and its distance to the activated neurons. TPM calcium imaging was performed simultaneously with electrophysiological recordings under isoflurane anesthesia (Figure 1). To locate the electrode, vasculature images at the cortical surface and ∼300–400 μm below the surface were obtained with OCT (Figure 1A). Imaging depth of TPM ranged between 180 and 250 μm below the cortical surface where strong activation of layer 2/3 neurons was observed (Figure 1C). Stimulation electrodes locate at 200–300 μm depth. In our preliminary experiment, at a stimulation frequency of 50 Hz, calcium signals quickly returned to baseline after the stimulation stopped for all the stimulation duration tested, i.e., 1, 5, 10, and 30 s (Figure 1D). In order to better study the onset and offset dynamics of neuronal responses, we kept the stimulation duration at 10 s for all trials, with 30 s of baseline calcium imaging and electrophysiological recording before and after stimulation (Figure 1B).

### Excitatory neurons’ temporal responses to electrical stimulation are frequency-dependent

**Figure 2:**
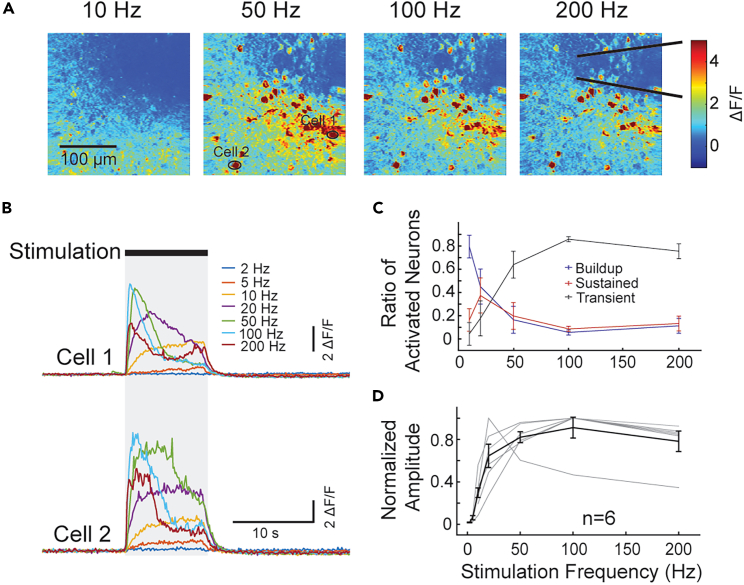
Stimulation-frequency-dependent neuronal calcium responses (A) Heat maps of amplitudes of calcium signals evoked by stimulation at frequencies of 10, 50, 100, and 200 Hz, 30 μA in a representative animal. The two black lines indicate putative boundaries of the electrode. Note that the biggest response was at 50 Hz. (B) Two example cells from (A) indicated by black circles. Calcium signals (ΔF/F0) evoked by electrical stimulations indicate three types of temporal responses at different frequencies, with buildup type responses evoked by low frequency (<20 Hz) and transient-type response evoked by high frequency (>50 Hz). (C) Quantification of the ratio of temporal response types at different frequencies. (D) Normalized peak amplitude of calcium signals evoked by different stimulation frequencies. Strongest responses were observed between 50 and 100 Hz. Gray lines represent individual animal. Data expressed as mean ± SEM.

Prior studies implementing TPM calcium imaging suggested different dynamics of responses to different electrical stimulation frequencies.^14^^,^^15^ In this study, we further examined how neurons respond to stimulation frequencies. First, we determined the neuronal activation threshold by ramping up stimulation current amplitude until at least one cell was visually activated at 50 Hz. Next, stimulations with a range of frequencies (2 Hz–200 Hz) at 10 μA above activation thresholds were delivered. The strongest activation was observed from stimulation frequencies between 50 Hz and 100 Hz (Figure 2A). Importantly, various temporal dynamics of calcium signals from the same neurons were also observed for different stimulation frequencies (Figure 2B). Low-frequency (<20 Hz) stimulation often elicits a calcium response described as a slow buildup, whereas high-frequency (>100 Hz) stimulation elicits a fast-rising and fast-decaying “transient” calcium response. Such temporal dynamics of calcium responses mimics the discharge patterns defined through the observations in other sensory systems, such as transient, buildup, and sustained.^26^^,^^27^^,^^28^ Transient responses are manifested as a brief discharge of action potentials only at the onset of stimulation, indicative of quick adaptation to the stimulation. Sustained and buildup responses are associated with constant or increased firing rate throughout the stimulation, exhibiting little or no adaptation. To quantify the number of different types of responses, we compared the response amplitude ratio of the first half of stimulation duration with the second half, with a ratio less than 80% qualitatively demonstrating a clear decay of calcium responses (transient), a ratio of more than 120% demonstrating a clear increase in calcium response (buildup), and a ratio between 80% and 120% showing similar magnitude of responses throughout the stimulation duration (sustained).

Below 10 or 20 Hz frequency, a large proportion of activated neurons were of the buildup type, whereas a large proportion of activated neurons were of the transient type when stimulation was at or above 50 Hz (Figure 2C). We also analyzed the peak amplitudes evoked by different stimulation frequencies (Figure 2D). The largest responses were observed between 50 Hz and 100 Hz for all animals except one, from which the strongest activation was evoked by 20 Hz stimulation. After that, responses started to decay with increases in stimulation frequency.

These results suggest that a range of 50–100 Hz frequency allows to achieve fast and efficient electrical stimulation effects.

### Excitatory neuron’s responses are also amplitude-dependent

**Figure 3:**
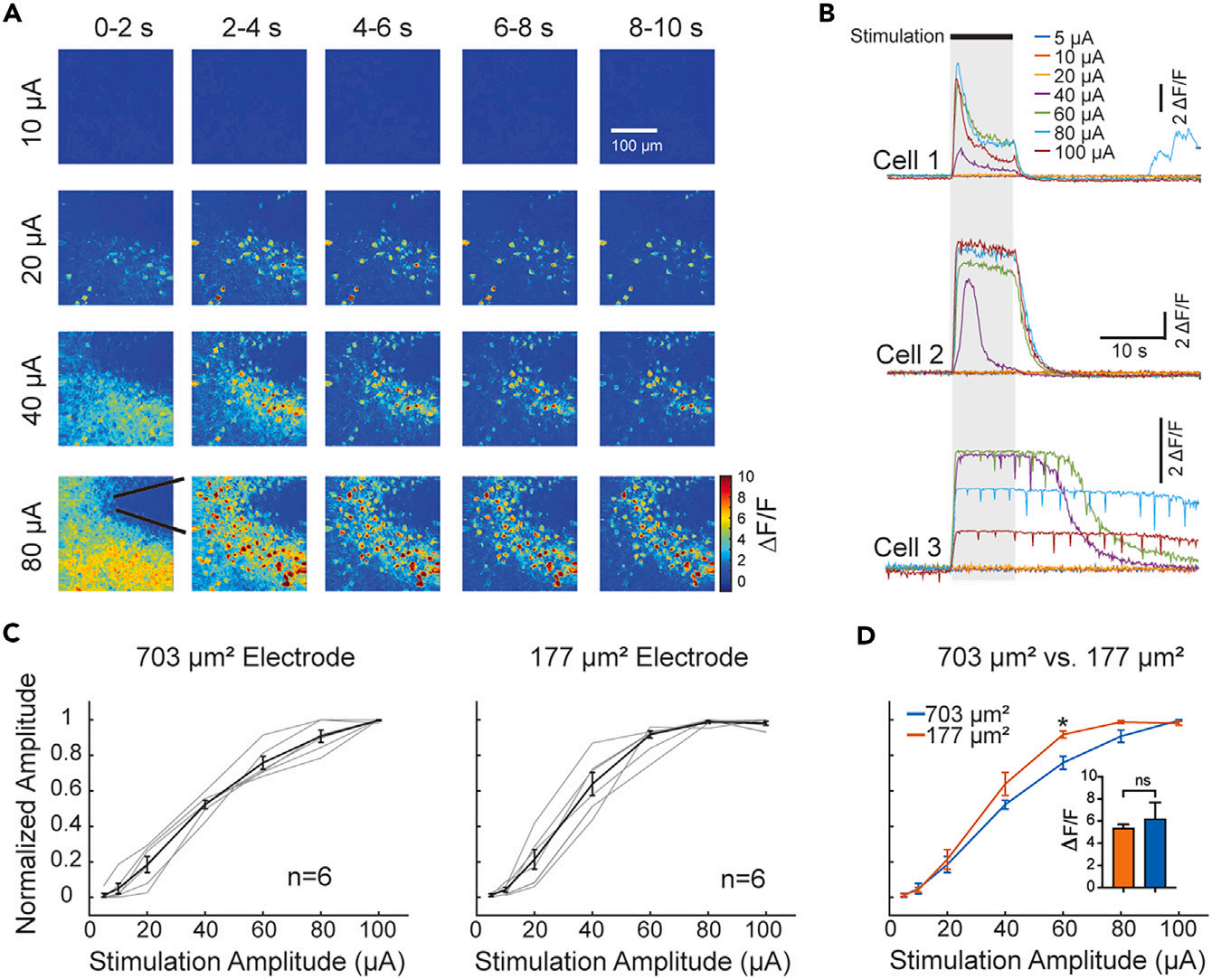
Stimulation-amplitude-dependent neuronal calcium responses (A) Heat maps of amplitudes of calcium signals evoked by different current amplitudes (top to bottom) at 50 Hz, averaged every 2 s during the 10 s of stimulation (left to right) from a representative animal. (B) Three representative cells show different temporal responses at different current amplitudes of stimulation. The long-lasting calcium signals after the end of the stimulation in Cell 3 suggest potential overstimulation at 80 and 100 μA. (C) Normalized peak amplitude of calcium signals evoked by different current amplitudes of stimulation from electrodes with geometrical surface area (GSA) of 703 μm^2^ (left) and 177 μm^2^ (right). Gray lines represent individual animal. Data expressed as mean ± SEM. (D) Comparison of calcium signals evoked by stimulation among electrodes with different GSA sizes. It appears that peak responses were reached with lower stimulation from smaller electrodes. However, no significant difference was observed in the peak calcium responses between 703 μm^2^ and 177 μm^2^ electrodes (inset, data shown as mean ± SEM, two-tail unpaired Mann-Whitney test, ∗p < 0.05, GraphPad Prism 9).

Prior studies using calcium imaging to investigate the effect of electrical stimulation at the cellular level focused on low stimulation amplitude.^14^^,^^15^^,^^18^ These studies significantly improved our understanding of the mechanisms of electrical stimulation; however, they did not address the relationship between stimulation amplitude and spatiotemporal dynamics of neuronal responses. Therefore, we examined the neuronal responses to different stimulation amplitudes, aiming to depict a stimulation-amplitude-dependent response curve and suggest a safe and effective range from acute electrical stimulations (Figure 3). As the strongest activation was evoked by stimulation frequencies between 50 Hz and 100 Hz, we stimulated cortical neurons by varying stimulation amplitudes from 1 μA to 100 μA with the frequency kept constant at 50 Hz (Figure 3A). Although in one animal a few neurons could be activated by a current amplitude as low as 2 μA, the activation threshold of most neurons and animals was above 10 μA, as represented by the three example cells in Figure 3B. Some cells showed linear relationships between calcium responses and stimulation amplitudes below 60 μA and saturated responses above 80 μA (Cell 1 and 2 in Figure 3B). Other cells showed calcium responses that lasted even after electrical stimulation ended, indicating potential overstimulation (Cell 3 in Figure 3B). Overall, a linear relationship between magnitude of calcium signals and stimulation amplitude was observed before reaching saturation level (Figure 3C), despite the size of stimulation electrodes (Figures 3C and 3D). No hydrolysis was observed with any of the tested parameters, suggesting water window of the electrodes were not exceeded.

### Excitatory neuron’s activation threshold and response type are spatial-specific

**Figure 4:**
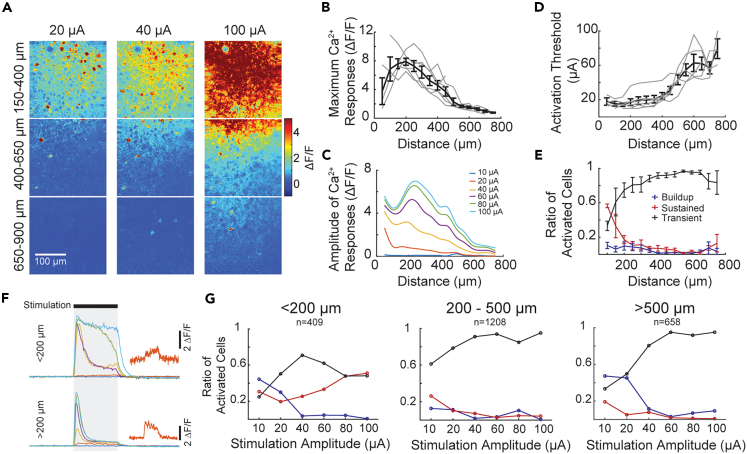
Spatial characterization of neuronal responses to electrical stimulation (A) Heat maps of calcium signals from TPM evoked by different current amplitudes of stimulation (20, 40, and 100 μA) at different locations away from the stimulation electrode (outside of the field of view on the top). (B) Neurons’ maximal calcium responses in relation to their distance to the stimulating electrode. Note that the responses peak at ∼200 μm away from the stimulating electrode and remarkably decrease at ∼500 μm away. (C) Amplitudes of calcium signals evoked by various levels of stimulation in relation to the distance to the stimulating electrode. Data are shown as LOWESS fitting curves (GraphPad Prism 9). (D) Activation threshold versus neuronal location relative to the stimulating electrode. Note that the activation threshold increases fast over 400 μm away. (E) Ratio of activated neurons with buildup, sustained, and transient types of calcium responses in relation to the distance to the stimulating electrode. (F) Amplitude-dependent dynamics of responses of two examples cells located at different distances from the stimulation electrode. The orange insets show zoomed in review of responses to 20 μA stimulation. (G) Ratio of neurons with buildup, sustained, and transient types of calcium responses in relation to stimulation amplitude at different distances from the stimulation electrode. Data are shown as mean ± SEM of six animals with a total of 2275 activated neurons (379 ± 53 neurons in each animal) analyzed in B, D, and E. Gray lines in B and D represent individual animal.

According to the above data, stimulation amplitude and frequency can determine the neuronal response magnitude and dynamics, which can reflect the sensation feature. Another important aspect for sensory perception is spatial discriminability, which determines how stimulation from different locations can be differentiated. Therefore, we also examined the relationship between neurons’ responses and the distance to the stimulating electrode (Figure 4A). We estimated the distance of individual neurons to the stimulation electrode according to x and y coordinates within each ROI as well as the relative x, y, and z distance of the ROI to the stimulating electrode according to OCT image. As might be expected, maximal responses of calcium signals decreased with the increased distance to the stimulation electrodes, and only minimal activation was observed beyond 800 μm away from the stimulating electrodes (Figures 4A and 4B). Stimulation levels near or slightly above the activation threshold, i.e., 20 μA, evoke calcium responses within a 500-μm radius from the stimulating electrodes, with the highest responses within 100 μm (Figure 4C). However, such spatial specificity diminishes with increased stimulation levels, with the peak response observed at approximately 200–300 μm away from the electrode when stimulation amplitude was above 60 μA (Figures 4B and 4C). The activation threshold of neurons within a 100-μm radius of the stimulation electrode is as low as 10 μA, whereas those 800 μm away from the stimulation electrode have an activation threshold of about 80 μA (Figure 4D). The threshold increases dramatically at a distance of more than 400 μm away.

We also examined the dynamic types of responses. In the vicinity of the stimulation electrode (<200 μm), a large proportion of activated neurons show the sustained type of calcium response; however, sustained response decreased with the increase in distance (Figure 4E). The proportion of transient responses, on the contrary, increased with increasing distance to the stimulating electrode (Figure 4E). In addition, we also observed a slight amplitude-dependency effect. For neurons more than 200 μm away from the electrode, approximately half of the cells were sustained or buildup at the activation threshold, and when stimulation level was at approximately 10 μA above the activation threshold, more than 80% cells had transient responses and remained the same up to 100 μA stimulation (Figures 4F and 4G). However, for neurons immediately next to the stimulation electrode (<200 μm), dynamic of cells changed from sustained to transient, then sustained with increase of stimulation amplitude (Figures 4F and 4G).

These results suggest potential different mechanisms underlying the activation of neurons in the areas proximal and distal to the stimulation electrodes, such as direct activation versus indirect activation.

### Post-stimulation suppression of neuronal activities and reduction of α-, β-, and γ-band power in local field potential

**Figure 5:**
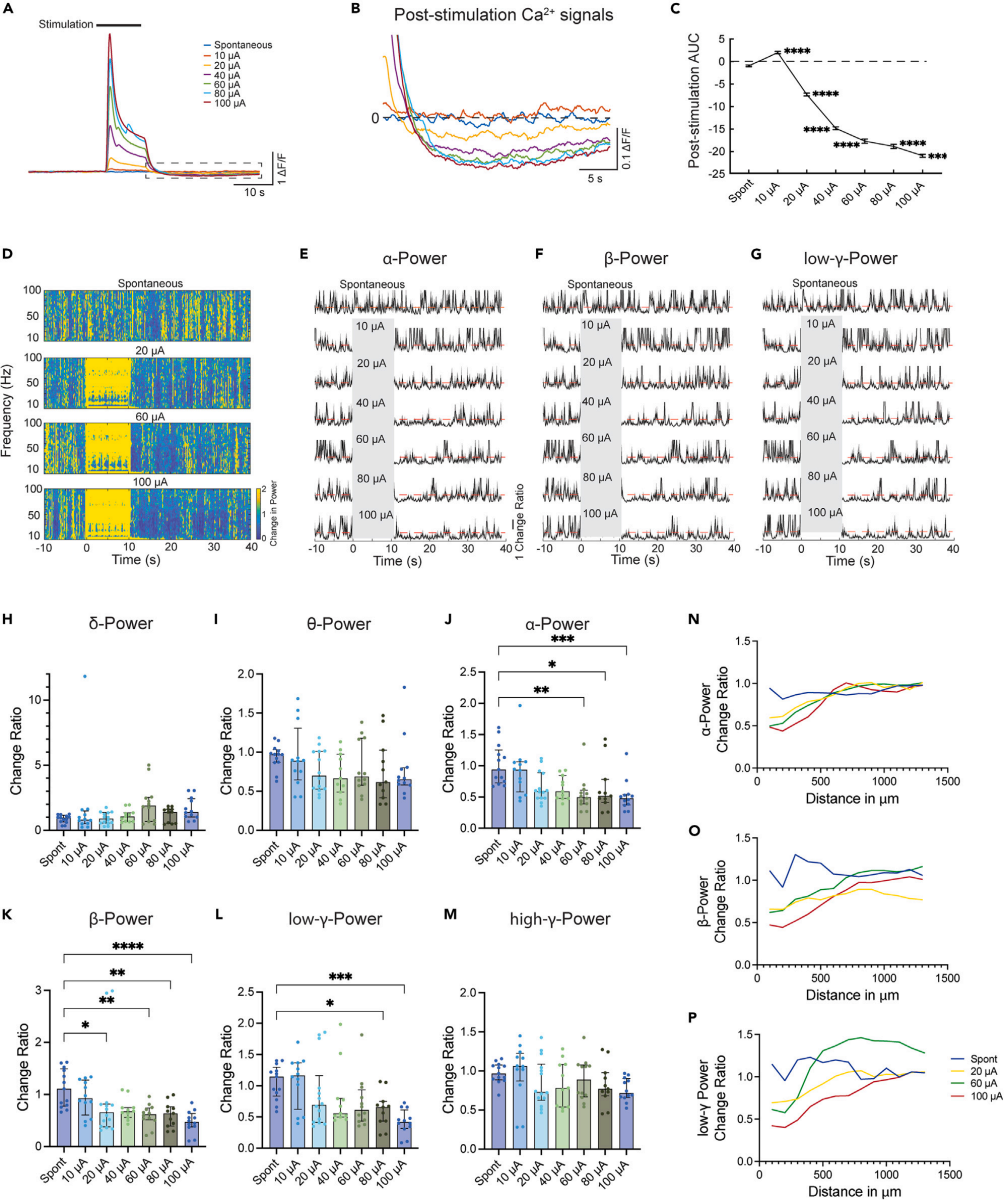
Post-stimulation suppression of neuronal activities (A) Calcium responses to different amplitudes of stimulation. Traces were averages of 2,275 cells from 6 animals with 703 μm^2^ electrodes implanted. (B) Enlarged view of post-stimulation calcium signals in the dotted box in (A) demonstrates an amplitude-dependent post-stimulation calcium signal reduction. (C) Area under the curve (AUC) of 5–30 s post-stimulation calcium signals. (Spont: spontaneous without stimulation; One-way ANOVA with Dunnett’s multiple comparisons test to Spont, ∗∗∗∗p < 0.0001, Graphpad Prism 9, data expressed as mean ± SEM). (D) Time-frequency power spectrum change ratio relative to the baseline. Heatmap is the average of 11–12 trials from 6 animals. Stimulation was delivered at 0–10 s. Note that the duration of reduction in power between ∼10 and 60 Hz appears to be positively correlated with the stimulation amplitude. (E–G) Power change ratio for α-, β-, and low γ-band across the recording. Gray areas indicate when stimulations were delivered. Orange lines are change ratio at 1, indicative of no change. (H–M) Ratio of post-stimulation to baseline power change for α- (J), β- (K), γ- (L and M), δ- (H), and θ-band (I) of LFPs at various current amplitudes recorded from electrodes located 100 μm away from the stimulating electrode. (Kruskal-Wallis test and Dunn’s multiple comparisons test, ∗p < 0.05, ∗∗p < 0.01, ∗∗∗p < 0.001, ∗∗∗∗p < 0.0001, GraphPad Prism 9, data expressed as median ± interquartile range). (N–P) Median value of the ratio of post-stimulation to baseline power for α- (N), β- (O), and low γ-band (P) at different distances away from the stimulating electrodes. Except for the power metrics showing significant changes in H–M at electrodes located 100 and 200 μm away, power changes recorded from electrodes further away were not statistically significant. Interquartile bars are not shown for clarity.

A previous study reported a localized, long-lasting stimulation-induced depression of neuronal excitability following prolonged high-frequency stimulation.^29^ A recent TPM calcium imaging study observed an about 17-s depression within 680 μm of the electrode after 30 s of continuous stimulation.^15^ In this study, we also observed that after electrical stimulation ended, calcium signals of activated neurons decay quickly below the baseline (Figures 5A and 5B). Quantification of the area under the curve (AUC) of post-stimulation calcium signals revealed that the suppression of baseline calcium signals is stimulation-amplitude-dependent, as the highest stimulation current evoked the largest suppression (Figure 5C). Although calcium signal decrease cannot indicate membrane hyperpolarization, nor a reduction in excitability, it suggests a reduction in firing rate.^30^

To further validate the post-stimulation effect on neuronal activity, we examined the impact of electrical stimulations on the global state of cortical activities. We recorded the local field potentials (LFPs) from the non-stimulating electrodes (up to 15) along the shanks of the probes. A wide-frequency band of LFPs demonstrated reduced power post-stimulation at the recording electrodes next to the stimulation electrode (100 μm away) in the time-frequency analysis (Figures 5D–5G). To statistically compare the change, we divided the average power of α-, β-, γ-, δ-, and θ-bands of LFPs after stimulations by that before stimulations. A significant amplitude-dependent power reduction was observed for the α-, β-, and low γ-bands, with higher amplitudes of stimulation currents inducing larger reductions, but not for high γ-, δ-, and θ-bands (Figures 5H–5M).

To delineate the spatial relationship between the change of LFP power and the distance to the stimulation electrode, we expanded our analysis to other electrodes, covering locations up to 1.3 mm away from the stimulation loci. The LFP power reduction in α-, β-, and low γ-bands did not demonstrate an apparent difference for electrodes located between 100 and 200 μm away from the stimulating electrode, whereas for electrodes located between 200 and 500 μm away from the stimulating electrode, we observed a negative relationship between the distance to the stimulation electrode and the degree of reduction. The reduction became indistinguishable from that of the non-stimulating trials over 500 μm away from the stimulating electrodes (Figures 5N–5P). However, it needs to be noted that these distinct responses between different electrodes can also stem from laminar effect (layer 2/3 versus layer 1). Unfortunately, with the single-shank Michigan style array, it is impossible to distinguish laminar and distance effect. Future studies with Utah or microwire designs can be used to further elaborate this.

### Relationship between excitatory neuronal responses and local E-field

**Figure 6:**
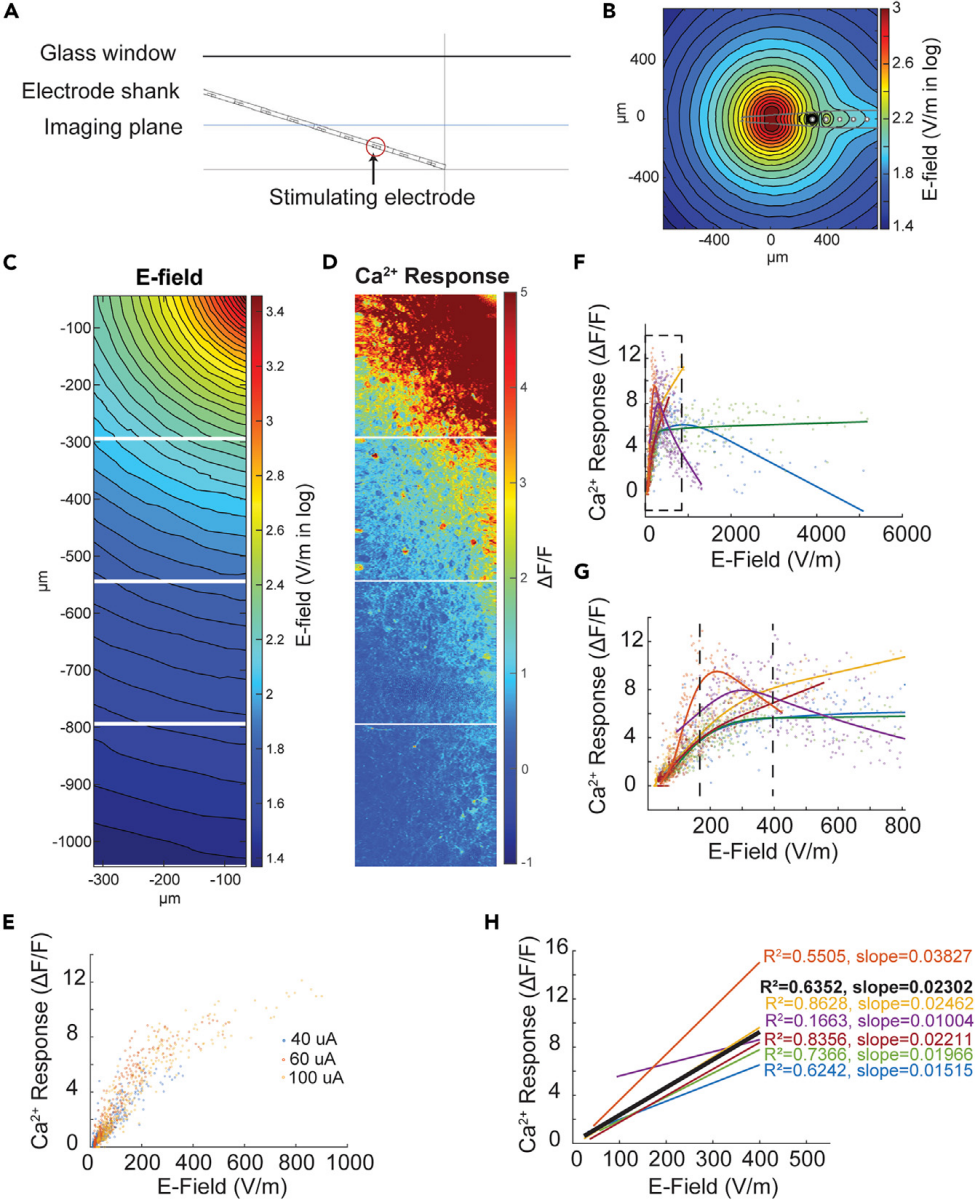
Relationship between neuronal responses and local electrical field (E-field) (A) E-field simulation configuration. Electrode shank angle and stimulation electrode depth were determined by OCT image. E-field at the TPM imaging plane was simulated. (B) An example E-field map of 100 μA stimulation at the TPM imaging plane with the center directly at the stimulation electrode in x and y locations. (C) E-field map of a 100 μA stimulation in the ROIs of TPM imaging of an example animal. x and y axes show the relative distance to the stimulating electrode. (D) Calcium responses to the electrical stimulation corresponding to (C). Note the alignment of E-field strength in (C) and calcium response amplitude in (D). (E) Relationship between calcium response and E-field at different stimulation amplitudes extracted from the example animal in (C) and (D). Regardless of the stimulation amplitude, the relationship between calcium response and E-field remains the same. (F) Summary of the relationship between calcium response and E-field of 6 animals at a stimulation level of 100 μA. Each color represents one animal. Solid lines are LOWESS fitting curves (GraphPad Prism 9). (G) Enlarged view of (F) to show that 4 out of 6 animals showed saturated calcium responses at around 400 V/m. One animal started showing saturation at about 175 V/m. (H) Linear regression demonstrated that below 400 V/m, calcium responses are linearly correlated to the E-field with a narrow range of slope. Each line represents one animal, and the black line is the linear regression analysis of all neurons from 6 animals.

Although the aforementioned analyses characterized the stimulation-amplitude- and frequency-dependent neuronal responses and their spatial properties, these results can be electrode-geometry-specific. This limitation precludes broader implementation of the results for new geometrical design or novel stimulation paradigms, e.g., simultaneous stimulation from multiple channels. Therefore, we performed E-field simulation around the stimulation electrode with a geometrical surface area of 703 μm^2^ and correlated the E-field with neuronal responses. We noticed that the amplitude of neuronal responses appears to be well correlated with E-field strength at 100 μA stimulation (Figures 6C and 6D). In line with our observation that neuronal responses can be saturated when stimulation amplitude is above 80 μA, when the E-field is above a certain level, neuronal responses start to decline or plateau. The saturation E-field level varies slightly among animals with 426 ± 89 V/m (mean ± SEM, n = 6 animals) (Figures 6F and 6G). However, below the saturation level, the relationship between calcium responses and E-field appears linear. To quantify this, we excluded data above 400 V/m and performed linear regression analysis. A slope of 0.02164 ± 0.0039 was revealed (Figure 6H).

To further verify the observation, we simulated the E-field at different stimulation amplitudes and plotted the calcium response amplitude against the E-field. Regardless of the stimulation amplitude, the relationship between calcium response and E-field remained the same for four out of six animals, manifested as similar linear correlation coefficients and saturation levels (Figure 6E). Two animals showed a shift in the linear correlation slope or the saturation level with different stimulation amplitudes. This may be related to the surrounding tissue condition, e.g., microbleeds or major vasculature presence.

This suggests that in addition to charge per phase or charge density, E-field can potentially be used as another index for evaluating the effect of ICMS. Although further studies are needed to establish a robust relationship between E-field and neuronal responses and to understand how surrounding tissue conditions, e.g., microbleeds or glial scars, and neuronal responses would affect the E-field distribution, our attempt shows promise.

## Discussion

### Effects of stimulation frequency on modulating neuronal activation

In the design of stimulation paradigms, stimulation frequency represents a critical parameter for clinical consideration, as it has been shown to modulate the intensity and quality of artificial perception in preclinical studies.^4^^,^^9^^,^^10^^,^^31^ A recent study also implied that different patterns of stimulation pulses may be needed for different therapeutic applications.^13^ As high frequency introduces larger cumulative charge injection when other variables in a stimulation paradigm are held constant, it is important to have a better understanding of how neurons respond to a wide frequency range from a regulatory perspective, for example, the lowest frequency that can activate neurons or the highest frequency that could jeopardize either the brain tissue or the electrode itself. Only within such frequency ranges can the therapeutic stimulation paradigms be determined to ensure the safety and performance of neurostimulation. Previously, only a limited set of stimulation frequencies were examined. In the current study, we examined a wider range of stimulation frequencies that have been proposed in preclinical and clinical studies. A weak activation of neurons was observed with 5 Hz stimulation around the activation amplitude threshold, whereas neuronal activation was rarely observed for stimulation frequencies below 5 Hz (Figure 1B). As other stimulation parameters, such as waveform, amplitude, and pulse widths, were kept constant, our results suggest that a temporal summation of charge will be needed to activate neuronal populations. When the current pulses were spaced out too much, the accumulated charge might not have been able to depolarize the cell’s membrane above their spike threshold to elicit the influx of calcium into the cells. Such temporal summation of charge and accumulated effects of electrical stimulation are also reflected by the “buildup” type of calcium responses observed during the entire course of stimulation, e.g., 10 s of stimulation in our study; gradually increased calcium signals were observed for stimulation frequencies between 5 Hz and 20 Hz. The strongest activation of neurons was observed between 50 Hz and 100 Hz of stimulation with sustained temporal responses, suggesting this range of frequency efficiently and reliably evoke neural activations. This also implies that adjusting the stimulation frequency can possibly modulate the temporal dynamics of the perceived sensation.

When a high frequency, e.g., above 200 Hz, was used, a transient temporal response could be characterized by a fast rise and fast decay of calcium signals. The biophysical or circuitry mechanism underlying such observations is unclear. However, this can explain some phenomena observed in clinical studies. For example, a prior study on visual restoration reported that increasing frequency of stimulation reduced the latency to the onset of phosphene detection, and increasing the stimulation duration did not necessarily increase the phosphene duration.^7^ The former can be explained by the high proportion of “buildup” neurons at low frequency and the latter by a “transient” response nature above 50 Hz stimulation. A recent study suggests that higher frequency (e.g., 100 Hz) stimulation recruits and synchronizes the activities of cortical inhibitory neurons, which suppresses the excitation of neurons via network inhibition during ICMS.^13^ However, high-frequency stimulation may also interfere with voltage-gated channels to reduce the excitability or calcium influx by shunting effects or other biophysical mechanisms.

### Safe threshold of neuronal activation may be within a narrow range of current amplitude

Currently, the Shannon equation based on charge density and charge per phase is widely quoted in determining the safety of electrical stimulation.^19^ However, this equation only applies to macroelectrodes for the far-field condition. For microelectrodes with a surface area <2000 μm^2^, a safety threshold of 4 nC/ph was suggested.^21^ However, this threshold may prevent the device from achieving its full effectiveness, as mounting evidence demonstrates the sensory or motor restoration threshold for ICMS is in the range of 3–10 nC/ph.^5^^,^^7^^,^^8^^,^^10^^,^^11^

In our study, although the minimal current amplitude that can activate neurons is 2 μA in a few neurons from one animal, corresponding to a charge per phase of 0.4 nC/ph, a majority of neurons were activated above 10 μA (2 nC/ph) (Figure 3C). A linear relationship was observed between the amplitude of evoked calcium signals and that of stimulation current for a 703 μm^2^ electrode, implying the predictability of neuronal responses to stimulation between 10 and 100 μA (2–20 nC/ph) in most cells. These observations appear to be consistent with previous studies with ICMS in different species.^5^^,^^10^^,^^11^^,^^32^ However, a few cells demonstrated prolonged activation even after the cessation of stimulation above 80 μA (16 nC/ph) (Figure 3B), suggesting potential overstimulation and excitotoxicity. This may explain how evoked sensations can last beyond the stimulation duration in some clinical investigations.^5^^,^^7^ Although the prolonged activation does not necessarily indicate immediate cell death, precautions are still needed when investigators propose to use high level of stimulation for chronic applications. In addition, the impact of high stimulation on the electrochemical properties of the electrodes themselves should be carefully evaluated in preclinical settings before using them in human studies.^33^

Another limitation of the 4 nC/ph threshold is that it does not take the electrode’s geometry into consideration. Our study suggests that although the activation thresholds for different size microelectrodes are comparable, neuronal responses can reach saturation levels at lower stimulation amplitude with smaller electrodes (Figures 3C and 3D). Thus, a safety profile of the stimulation threshold should be determined not only by the stimulation amplitude but also by the electrode’s geometrical property and charge limit. In addition to electrode size and shape, stimulation pulse shape and source location can potentially affect neuronal responses to electrical stimulation. Efforts such as ours to correlate the neuronal responses with the E-field could potentially mitigate these limitations. Indeed, we observed a consistent linear correlation between E-field and magnitude of neuronal responses below the saturation level (Figure 6H). Although the relationship needs to be further validated with different types of electrodes, this attempt demonstrates promise for using E-field as an additional index to assess the stimulation effect.

### Spatial specificity of frequency- and amplitude-dependent neuronal activations

Damage to the brain tissue and neurons in the vicinity of implanted microelectrodes is inevitable^34^^,^^35^ and could be observed from persistent fluorescent signals of neurons close to the electrode in our study as well (Figure 1A). The evaluation of those neurons proximal to the electrode could be less meaningful, as the innervation from other cortical neurons could have already been severed. Thus, analysis of the spatial profile of neuronal responses is needed to infer the safety and effectiveness of electrical stimulations. Because a distance of 50 μm from the microelectrode can be considered a point source during electrical stimulation from modeling studies, the strength of the electrical field at any specific location >50 μm is distance-dependent.^21^^,^^36^

We estimated the distances of all identifiable neurons under TPM and associated them with temporal response types, activation thresholds, and amplitudes. An interesting phenomenon is the inverse relationship between the percentages of sustained and transient types of responses in relation to neurons’ distance to the stimulating electrode. A larger proportion of activated neurons near the stimulating electrode showed sustained temporal responses to electrical stimulation above the activation threshold, whereas a larger proportion of activated neurons away from the stimulating electrode showed transient responses. The mechanisms underlying the percentage change of sustained and transient type responses at the locations away from the stimulating electrodes are unclear.

One possibility is that within 200–300 μm, neurons can be directly activated by electrical stimulation, which could be associated with the continuous exposure to the electrical field distribution during the stimulation, whereas the neurons >300 μm away from the electrode are primarily activated by indirect connections from those in the vicinity of the stimulating electrode and are susceptible to the recruitment of delayed inhibition in the neural circuitry. Previous studies investigating the synaptic effect on the electrical-stimulation-evoked neuronal activity showed controversial results. Earlier *in vivo* calcium imaging work suggested that direct excitation of neurons is the major activation mechanism at low stimulation levels.^18^ However, later work found that blocking the glutamatergic input increased the activating threshold, suggesting a strong synaptic effect.^37^ Thus, further circuit level study and analysis will be needed to tease apart the underlying mechanisms of distance-related response types.

Not surprisingly, the activation threshold increases with the increased distance of neurons to the stimulation electrode (Figure 4B). However, the amplitude of calcium signals evoked by electrical stimulations reached the maximum at the location 200–300 μm away from the stimulating electrode (Figure 4C). It is not clear why the strongest calcium signals seemed not to appear in the immediate vicinity of the electrode, but it may be attributed to the damaged neural networks surrounding the implanted electrodes or different activation thresholds of excitatory and inhibitory neurons in the same electrical field. Unfortunately, in Thy1-GCaMP6s animals used in the study, only a subset of excitatory neurons is expressed with calcium sensor. Further studies are needed to understand the activation thresholds and biophysical properties of inhibitory neurons, as well as the role of interactions between excitatory and inhibitory neurons in shaping the spatiotemporal specificity of responses to stimulation, and the degree of implantation damage to both neuronal subtypes.^13^^,^^38^^,^^39^

### The effects of ICMS on the cortical states

Electrical stimulations have been used to alter the cortical and behavioral states in animal studies.^40^^,^^41^ The application of ICMS has also been reported to induce persistent but reversible depression of neuronal excitability.^29^^,^^42^^,^^43^ As α-, β-, and γ-band cortical waves are associated with varying degrees of attention, and δ- and θ-band waves with deep relaxation and sleep, our data suggest that even a short period of electrical stimulation above the activation threshold could impact attention.^44^^,^^45^^,^^46^^,^^47^ This implies that any future indication for use of an electrical stimulation paradigm should consider the potential side effects that may impact the benefit-risk profile of the application. However, one caveat in our current study is that because the animals were under isoflurane anesthesia during the experiments, the confounding and interactive effects from such anesthetic agents on the cortical states cannot be easily teased apart.^48^^,^^49^^,^^50^ A previous study indeed noticed increased threshold for inducing motor activity through ICMS with anesthesia of diazepam, which shares a similar anesthetic mechanism by enhancing inhibitory GABAergic transmission.^51^^,^^52^ In future studies, a confirmatory experiment could be done in awake animals.

The high repeatability and spatiotemporal resolution of neuronal responses evoked by electrical stimulation suggest that the future design of stimulation parameters should be centered around a narrow amplitude range between activation and saturation thresholds, as well as a frequency range optimal for eliciting proper temporal responses. These responses could ensure maximal spatiotemporal specificity for the desired effects and prevent unwanted non-specific effects in the therapeutic domain.

### Limitations of study

#### Neuronal responses to electrical stimulation from microelectrodes with different GSAs

In our early pilot studies, we used microelectrodes with smaller geometrical surface areas (GSAs), i.e., 177 μm^2^. Although we were able to obtain calcium responses from neurons activated by electrical stimulation (i.e., at different current levels at 50 Hz, Figures 3C and 3D), electrode failure was observed for all tested animals when higher frequencies of electrical stimulation were applied. This could be related to the total charge delivered from the smaller electrodes, as was evident in our bench testing using cyclic voltammetry (data not shown). Thus, we were not able to obtain neurons’ calcium responses to various higher frequencies of electrical stimulation delivering much larger total charges to the electrode with smaller GSAs (i.e., 177 μm^2^ electrodes). Also, the spatial relationship between the electrical stimulation and calcium responses could not be derived. Future experiments investigating frequency-dependent neuronal responses should restrict the total charge delivered to the electrode.

#### Spike activities affected by electrical stimulation

The firing of neurons during the electrical stimulation could not be obtained from our current configuration, as strong electrical artifacts made separating the actual spikes impossible. We noticed that the firing before and after the electrical stimulation was sparse, possibly due to anesthesia.^49^^,^^50^ As our stimulation parameters covered a wide range of current levels and frequencies, we were not able to generate a traditional raster plot used to extract a spike-timing histogram due to limited repetition for each stimulation setting. Thus, future experiments with increased repetition trials and fixed stimulation parameters on awake animals receiving electrical stimulation could provide further evidence of the impact of electrical stimulation on neurons’ firing pattern changes.

#### Detection artifacts of calcium responses evoked by electrical stimulation

Although calcium imaging is a very powerful tool for visualizing real-time neuronal responses, making accurate inferences of action potentials from calcium imaging is challenging. Some simultaneous spike recording and calcium imaging studies suggest a linear correlation between ΔF/F and the number of spikes,^53^^,^^54^^,^^55^ whereas others imply non-linearity.^55^ Another complication is that the quantification of ΔF/F depends on the baseline. In our study, we observed the maximal responses at 200 μm away from the stimulating electrode rather than in immediate proximity to the electrode. This could be due to implantation injury caused by network disruption, as discussed earlier, and could also be an analysis artifact. Baseline fluorescent signals next to the electrodes can be higher due to the presence of dead cells, artificially leading to a lower ΔF/F.

#### Laminar differences of neuronal activation by electrical stimulation

Studies have found that the thresholds to induce a behavioral response were layer specific, with higher threshold in layer 2/3 compared with layer 5.^12^^,^^52^^,^^56^ In our study, we only focused on the layer 2/3 neurons due to the imaging depth limitation inherent to the TPM and the implantation strategy in which probes were inserted into the cortex with an angle to allow for the visualization of neurons next to the electrode through the window. The stimulation was provided by the electrodes close to the distal end of the probes at the depth of 200–300 μm. These neurons play important roles in integrating information across different cortical regions and layers by making corticocortical and interlaminar connections.^57^^,^^58^ Current widely used Utah array or custom-made arrays have electrode tip ended at about 1.5–2 mm depth, which can possibly stimulate layer 2/3 neurons in human brain depending on the region.^7^^,^^59^ Meanwhile, the development of novel arrays with electrodes aligned along the electrode shank makes the stimulation of different layers of the cortex possible. Therefore, further studies will be needed to understand the response properties in the deeper locations, especially those larger pyramidal neurons in the layer 4 and 5 that directly receive or send projections to other regions of the brain.

## STAR★Methods

### Key resources table

**Table:** 

REAGENT or RESOURCE	SOURCE	IDENTIFIER
**Experimental models: Organisms/strains**		

C57BL/6J-Tg(Thy1-GCaMP6s) GP4.3Dkim/J	Jackson Laboratory	Stock No: 024275

**Software and algorithms**		

Fiji: ImageJ	Fiji	http://fiji.sc
MATLAB R2021a	MathWorks	https://www.mathworks.com/products/matlab.html
LabVIEW	National Instruments	https://www.ni.com/en-us/shop/labview.html
Trellis (v. 1.14)	Ripple LLC	https://rippleneuro.com/support/software-downloads-updates/
Graphpad Prism (V9)	GraphPad	https://www.graphpad.com/scientific-software/prism/
Comsol Multiphysics (Ver. 5.8)	Comsol	https://www.comsol.com/

**Other**		

Scout (Grapevine Processor)	Ripple LLC	https://rippleneuro.com/ripple-products/
Nano2+Stim front end	Ripple LLC	https://rippleneuro.com/ripple-products/
Microelectrode	NeuroNexus	A1x16-3mm-100-703-CM16LP
Microelectrode	NeuroNexus	A1x16-3mm-50-177-CM16LP

### Resource availability

#### Lead contact

Further information and requests for resources and reagents should be directed to and will be fulfilled by the lead contact, Meijun Ye (Meijun.Ye@fda.hhs.gov).

#### Materials availability

This study did not generate new unique reagents.

### Experimental model and study participant details

All procedures were approved by the FDA White Oak Institutional Animal Care and Use Committee and comply with the National Institutes of Health Guide for the Care and Use of Laboratory Animals. Adult male Thy1-GCaMP6s transgenic mice C57BL/6J-Tg(Thy1-GCaMP6s) GP4.3Dkim/J (Jackson Laboratory, Bar Harbor, ME, Stock No: 024275) were used in the study. Mice were 2–4 months old at the time of window surgery and electrode implantation.

### Method details

#### Surgery

During surgery, mice were anesthetized with a 4% induction dose of isoflurane (Henry Schein, Melville, NY), and then positioned in a stereotaxic apparatus (David Kopf Instruments, Tujunga, CA). Mice were maintained under anesthesia with 1–1.5% isoflurane (0.8 L∕min O2), body temperature was maintained at ∼37°C with a thermostat-controlled heating plate (Model TC-1000, CWE Inc., Ardmore, PA), and respiration rate was monitored and maintained at ∼100 breaths∕min during the procedure. A craniotomy (∼2 × 3 mm) was administered over the left motor and somatosensory cortex (coordinates relative to bregma in mm: AP −0.5 to −3.5, L 0.5 to 2.5) using a high-speed dental drill (Osada, 0.25-mm drill bit, Osada, Inc Los Angeles, CA). A custom-cut 2 × 2 mm glass coverslip (sterilized #0) was placed on the surface of the cortex above the dura and attached to the skull with Kwik-Sil (WPI, Sarasota, FL) and dental cement (Parkell C&B Metabond, Edgewood, NY) on three sides. A single-shank, 16-channel, Michigan-style microelectrode array (A1X16-3mm-50-177-CM16LP or A1X16-3mm-100-703-CM16LP, NeuroNexus, Ann Arbor, MI) was inserted into the cortex with an angle of 10–20° relative to the brain surface through the posterior edge of the craniotomy without Kwik-Sil by a motorized micromanipulator (Siskiyou, Grants Pass, OR) to the approximate depth of ∼200 to 300 μm and with the array tip terminated in the middle of the glass coverslip (approximate coordinates: AP -1.5, L 1.5). Our prior study demonstrated that the insertion of electrodes at an angle severed dendrites.^60^ Therefore, the electrode array was inserted with the electrode facing down to minimize the impact of tissue damage on the interpretation of the stimulation evoked responses, as up-facing electrode would primarily stimulate severed dendrites rather than cell soma and axon hillock. The exposed brain at the posterior edge of the craniotomy was covered with a thin layer of Kwik-Sil after electrode insertion, and the glass window and percutaneous electrode array connectors were adhered to the skull permanently with dental cement. A stainless-steel ground pin (Fine Scientific Tools, Foster City, CA) was anchored through a burr hole drilled posterior to the lambdoid suture contralateral to the craniotomy as a common reference. A custom-made metal bar with a screw notch was attached over the skull on the right hemisphere for stabilizing the head during *in vivo* imaging, which was performed one day after the surgery.

#### OCT and TPM imaging

As previously reported,^60^ animals were imaged on a custom-built imager with TPM and OCT channels in which the TPM (970 nm) and OCT (1,317 nm) beams were colinear. For the imaging protocol, mice were anesthetized with a 4% induction dose of isoflurane and then maintained with 1%–2% of isoflurane throughout imaging and stimulation sessions. Body temperature was maintained at 37°C on a heating pad. The animal was positioned for OCT and TPM imaging using a three-axis motorized animal stage (AS) (Thorlabs Inc., Newton, NJ). Reflectance, angiography, and flow videos were acquired and processed in near real time using custom software written in LabVIEW (National Instruments, Austin, TX), MATLAB (Mathworks, Natick, MA), and C/C++ and using the CUDA parallel processing platform (NVIDIA, Santa Clara, CA) on the system computer’s Tesla K40 GPU (graphical processing unit) video card (NVIDIA). The reflectance video used standard OCT processing, taken with a pixel density of 4 μm/pixel, was two 2 × 2 mm scans (500 × 500 lateral pixels, excluding flyback) covering the entire window with the focus just beneath the window (OCT surface: at ∼0- to 50-μm deep) and ∼300- to 400-μm deep (OCT deep, Figure 1A), the latter roughly at the depth of the electrode tip.

TPM imaging was performed immediately following OCT imaging. The TPM channel uses a Mai Tai titanium:sapphire femtosecond laser source (Spectra-Physics, Santa Clara, CA) tuned to a center wavelength of 920 nm with 100-fs pulse duration for excitation. A 20× water immersion objective (Nikon, Tokyo, Japan) with a numerical aperture (NA) of 1 was used for TPM imaging. The OCT channel served to guide subsequent two-photon imaging (Figure 1A). Because the two-photon and OCT beams are aligned colinearly, the lateral position of the two-photon field of view (FOV) on the cortex could be quickly identified after OCT imaging. An overlay on the OCT face image enabled rapid location of the electrode and the estimation of distance between electrode and TPM imaging ROIs, where the 2 × 2 TPM montage could then be immediately collected. For calcium imaging, image videos were taken at an 8-fps frame rate in the vicinity of electrodes (Figure 1A).

#### Electrical stimulation, calcium imaging, and electrophysiology

Electrical stimulation and electrophysiological recording were performed by a Scout system and Nano2+Stim front end (Ripple Neuro, Salt Lake City, UT). For each animal, each stimulation paradigm was repeated four to five times in a random order. Electrophysiological signals were acquired with a 30 kHz acquisition rate. The recording was triggered by the start of TPM calcium imaging via customized LabView program to synchronize stimulation, electrophysiological recording, and calcium imaging. The electrode at approximately the Layer II/III cortex was selected to deliver charge-balanced biphasic cathodic leading stimulations, with a pulse width of 200 μs and an inter-pulse interval of 100 μs. Stimulation amplitude ranged from 1 μA to 100 μA, whereas the frequency ranged from 2 Hz to 200 Hz. An average of 4 regions of interest (250 μm × 250 μm) with different distances to the stimulation electrode were located for imaging. Stimulation began once the depth of the first location was finalized. A TPM video was acquired for each stimulation trial. The imaging size was 250 μm with a resolution of 1 μm/pixel. Imaging depth ranged between 180 and 250 μm below the cortical surface as the strong activation of neurons were observed (Figure 1C). In our preliminary experiment, at stimulation frequency of 50 Hz, calcium signals quickly returned to the baseline after the end of stimulation for all the stimulation duration (Figure 1D). Thus, the stimulation duration was kept at 10 s for all trials with 30 s of baseline imaging/recording before and after stimulation (Figure 1B). The impedance was checked both before and after the stimulation session to ensure the stimulating electrodes were viable. When testing the effect of one parameter, the other parameters were held constant.

#### Data analysis

Band-pass (0.5–300 Hz) filtered local field potentials (LFP) were compared before and after electrical stimulations. MATLAB code, *pspectrum*, was used to estimate the power spectral density (PSD) before and after electrical stimulation. PSD change ratio was determined via dividing PSD at any time by that before stimulation. To assess the statistical significance of the change, we averaged the power across 10 s of post-stimulation recording (starting from 2 s after stimulation to exclude stimulation artifacts) and divided that by the power before stimulation. The power bands are defined as δ: 1–4 Hz, θ: 4–8 Hz, α: 8–13 Hz, β: 13–20 Hz, low-γ: 20–56 Hz, high-γ: 64–100 Hz.

For calcium imaging analysis, we used ImageJ to manually identify the individual neurons and to extract fluorescent intensity of calcium signals. Electrical stimulation-evoked neuronal activity was quantified by full field or individual neuron fluorescent calcium signals. Calcium responses were quantified by ΔF/F, the change in fluorescent intensity calculated as (Ft-F0)/F0, where F0 is the averaged baseline fluorescent intensity during 10 s before electrical stimulation (Figure 1C). MATLAB code was utilized to identify activated cells, amplitude of calcium responses, temporal response types (build-up, sustained, and transient), and the distance to the stimulating electrodes based on the cell coordinates. Quantitative analysis was performed to understand the relationship between individual stimulation parameters, (e.g., frequency, amplitude), relative location of the cell to the electrode, and neuronal responses, (e.g., temporary dynamics, peak amplitude of calcium responses).

To determine whether a neuron was activated by electrical stimulation, we compared ΔF/F values during the 10 s before stimulation to that during stimulation using one-tailed two sample Kolmogorov-Smirnov test (K-S test) with MATLAB function, *kstest2*, with α set at 0.002. We tried different methods to determine the activation of neurons, including using 2 or 3 standard deviations of the baseline signal. The K-S method provided the best alignment with visual determination of activation status of a subset of neurons. Temporal dynamic type of calcium response was determined by dividing the average ΔF/F during the first 5 s of stimulation by that of the second 5 s. Amplitude of calcium responses was defined as the peak ΔF/F during stimulation.

#### Electrical field (E-field) simulation

COMSOL Multiphysics software was used for Finite element modeling of the 703 μm^2^ implanted electrodes in brain tissue. The CAD image of A1x16-5mm-100-703 NeuroNexus electrode with 16 electrodes was imported in the COMSOL software and was located according to the OCT images of the *in vivo* animal study. Each electrode had a cylindrical shape with a 30-μm diameter and a 7.5-μm thickness located inside a non-conductive shaft of 15-μm thickness, facing away from the tissue top surface. The electrical properties of iridium in the COMSOL database were used for electrodes. A biphasic pulse with 20–100 μA current was applied to the active electrode, and other electrodes were modeled with floating boundary conditions. The brain tissue was modeled as semi-infinite uniform media with 0.4 S/m conductivity and relative primitivity of 1. For ground, a conductive cylinder with 2.2 mm diameter was placed at x = 3700 μm and y = 2500 μm distance from the tip of the electrode shaft based on the animal *in vivo* experiments. After simulation using an AC/DC module, the voltage and electric field were calculated at the same geometry locations as those of calcium images captured in the *in vivo* animal study.

### Quantification and statistical analysis

All statistical analyses were performed in Graphpad PRISM 9.0. All sample sizes and animal numbers are denoted in the respective figure legends and/or captions. To compare the amplitude dependent calcium responses between 703 μm^2^ and 177 μm^2^ GSA electrodes, two-tail unpaired Mann-Whitney test was used. One-way ANOVA with Dunnett’s multiple comparison test was used to compare the AUCs of post-stimulation calcium responses among different stimulation amplitudes. To compare LFP power change, Kruskal-Wallis test and Dunn’s multiple comparison test was used. Linear regression analysis was used to determine the relationship between E-field strength and amplitude of calcium responses.

## Data Availability

The original data reported in this paper will be shared by the lead contact upon request. This paper does not report original code. Any additional information required to reanalyze the data reported in this paper is available from the lead contact upon request.
